# Unveiling the Dark: Exploring the Nomological Consistency of the Short Dark Triad and Dirty Dozen Scales

**DOI:** 10.5964/ejop.12591

**Published:** 2025-05-28

**Authors:** Marco Tommasi, Marco Lauriola, Aristide Saggino

**Affiliations:** 1 Department of Psychology, Università degli Studi “Gabriele d'Annunzio” Chieti-Pescara, Chieti-Pescara, Italy; 2 Department of Social and Developmental Psychology, “La Sapienza” University of Rome, Rome, Italy; Lancaster University, Lancaster, United Kingdom

**Keywords:** Dark Triad, nomological pattern, narcissism, personality, social disinhibition

## Abstract

We examined the consistency of the Short Dark Triad (SD3) and the Dirty Dozen (DD) scales, which are widely used for assessing Dark Triad traits (Machiavellianism, narcissism, and psychopathy), in a community Italian sample of 504 individuals aged 18 to 89 years. The findings revealed strong convergence for Machiavellianism and psychopathy across the two scales. In contrast, narcissism demonstrated weaker convergence, with moderate correlations between the SD3 and DD scales. Nomological consistency, the degree to which different indicators of a construct share similar associations with external criteria, was assessed using four sets of criteria: psychopathy or empathy, the Five-Factor Model of personality, mental health (psychological well-being, anxiety, and depression), and social disinhibition. Both scales showed moderate consistency with empathy, the Big Five, and social disinhibition criteria but displayed inconsistency concerning mental health criteria. Psychopathy and Machiavellianism exhibited distinct patterns in relation to social disinhibition. Narcissism demonstrated the most divergence from other traits and the highest inconsistency between SD3 and DD. The SD3 appeared to focus predominantly on grandiose narcissism, whereas the DD scale likely encompasses both grandiose and vulnerable aspects of narcissism. Consequently, SD3 and DD cannot be considered fully equivalent measures of Dark Triad traits.

The term “Dark Triad” refers to a personality structure comprising Machiavellianism, narcissism, and psychopathy (Paulhus & Williams, 2002). Machiavellianism entails manipulativeness, cynicism, and a strategic interpersonal orientation (Christie & Geis, 1970). Narcissism involves grandiosity and dysfunctional self-esteem strategies (Jones & Paulhus, 2014), while psychopathy is characterized by lack of empathy, impulsivity, and antisocial behavior (Jones & Paulhus, 2014).

The three components of the Dark Triad are subclinical traits, and research suggests that Machiavellianism and psychopathy can be considered interchangeable (O’Boyle et al., 2015; Vize et al., 2018; Watts et al., 2017). However, narcissism measured with different scales does not consistently converge within the same trait (O’Boyle et al., 2015; Watts et al., 2017). This lack of consistency may be attributed to the different scales used to measure these personality traits (Watts et al., 2017).

The Short Dark Triad (SD3; Jones & Paulhus, 2014) and the Dirty Dozen (DD; Jonason & Webster, 2010) are commonly used self-report scales to measure Dark Triad traits (Maples et al., 2014). Studies comparing the psychometric properties of these scales suggest that SD3 has higher validity than DD (Gamache et al., 2018; Geng et al., 2015; Maples et al., 2014). Notably, the narcissism scales of SD3 and DD show a weak correlation, possibly due to SD3 measuring grandiose narcissism while DD assesses both grandiose and vulnerable narcissism (Maples et al., 2014). Narcissism can manifest as grandiose or vulnerable forms, characterized by different traits (Gore & Widiger, 2016).

One of the major problems in psychological research is the *jingle-jangle fallacy*. This fallacy consists of conceptual mistakes that happen when psychological constructs are confused in relation to their labels or names. In particular, jingle fallacy happens when two psychological scales use the same name for indicating psychological constructs that are different (e.g., two scales that measure motivation, but one measure intrinsic motivation while the other measures extrinsic motivation); jangle fallacy happens when two scale have different names, but they are measuring the same construct (e.g., a scale of “self-efficacy” and a scale of “self-confidence” that are measuring the same construct). The negative consequences of the presence of jingle-jangle fallacy are theoretical confusion, difficulty to discriminate between constructs and, therefore, to create predictive models and the risk to make wrong generalizations or conclusions from empirical data. In literature, for example, some authors (Marsh et al., 2019) found that the constructs “self-efficacy” and “self-concept” are confused and overlapped. Therefore, a nomological analysis, through Multi-Trait Multi-Method analyses can help researchers to improve the discrimination between constructs and to increase their theoretical and empirical utility (Marsh et al., 2019). Scales with the same name but with different degrees of convergence and divergence with other measures probably assess different constructs. In the case of Dark Triad traits, one could argue that both SD3 and DD might use the same labels for Machiavellianism, narcissism and psychopathy scores, yet they could be assessing different constructs.

Apart from convergent and discriminant analyses, additional criteria such as prediction consistency and nomological consistency are essential for assessing the consistency of Dark Triad traits (Thielmann & Hilbig, 2019). Prediction consistency evaluates whether SD3 and DD scales predict the same outcomes or behavioral criteria, highlighting their substantial interchangeability in research or applied contexts. On the other hand, nomological consistency examines the overall pattern of correlations between the scales and other relevant constructs, ensuring that these correlations are consistent with theoretical expectations (Thalmayer et al., 2011). Therefore, prediction and nomological consistency offer a comprehensive framework for assessing whether SD3 and DD accurately measure the same underlying traits or represent distinct conceptualizations. To assess prediction and nomological consistency of SD3 and DD, we collected data from a community sample of individuals without psychological syndromes, as Dark Triad traits should manifest consistently even among the general population (Fleeson & Noftle, 2009).

In this study, we aimed to test the prediction consistency of SD3 and DD by comparing their prediction consistency with four sets of psychological variables. Previous research has linked Dark Triad traits to maladaptive behavior, substance abuse, and mental health variables (Azizli et al., 2016; Egan et al., 2014; Muris et al., 2017; Stenason & Vernon, 2016). Additionally, Dark Triad traits have been connected to the Five-Factor Model (FFM) of personality, with conscientiousness and agreeableness negatively associated with psychopathy and Machiavellianism, and openness and extraversion positively associated with narcissism (O’Boyle et al., 2015). Considering the potential overlap between Machiavellianism and psychopathy, their connection with disinhibition was explored, as psychopathy shows a positive relation with disinhibition while Machiavellianism exhibits a moderate association with self-control (Jones & Paulhus, 2014; Miller & Lynam, 2015).

To evaluate the nomological consistency of SD3 and DD, we examined their relationships with four sets of variables: psychopathy and empathy, FFM personality traits, mental health indicators, and disinhibition versus constraint measures. By investigating these variables, we aimed to gain a comprehensive understanding of the consistency of Dark Triad traits and their associations across different constructs.

## Method

### Participants and Procedure

A total of 504 Italian participants (58% females) with ages ranging from 19 to 89 years (*M* = 40.79; *SD* = 20.62) took part in the study. Most participants were either full-time or part-time students (37.6%), who were recruited for the study by the first author of his paper while they were attending university courses. The remaining participants were non-students employed in various occupations such as dependent workers (13.5%), housewives (12.7%), retired individuals (12.1%), employees (4.2%), freelance professionals (4%), trade officers (3.8%), teachers and professors (2%), soldiers and military personnel (1.6%), farmers (0.6%), seasonal or unskilled workers (1.4%), managers or business owners (0.8%), and drivers (0.4%). These participants were contacted through announcements or by word of mouth. Using GPower, we estimated the minimum required sample size for linear regression models with 3 predictors, setting α = 0.05, 1 – β = .80 and medium effect size *f* = .15. The calculation indicated that the sample should consist of at least 43 participants. The participants completed pencil-and-paper questionnaires administered by the same expert examiner. Informed consent was obtained from all participants, and their participation was voluntary. The study followed the ethical principles outlined in the Declaration of Helsinki and was approved by the regional ethical committee for biomedical research (Ref: rich99n3w). Material used for this study and R script codes for the analyses are available at Tommasi (2025).

### Measures

#### Dark Triad Traits

The Short Dark Triad (SD3) scale, consisting of 27 items measured on a five-point Likert scale, assessed the components of Dark Triad: Machiavellianism, Narcissism, and Psychopathy. The Dirty Dozen (DD) scale, consisting of 12 items measured on a seven-point Likert scale, also assessed the same Dark Triad facets. The Italian adaptations of the DD (Schimmenti et al., 2019) and the SD3 (Somma et al., 2019) were used in this study.

#### Psychopathy and Empathy

The Levenson Self-Report Psychopathy Scale (LSRP; Italian adaptation: Somma et al., 2014) comprised 26 items measured on a four-point Likert scale. It yielded subscales for Primary Psychopathy and Secondary Psychopathy. The Balanced Emotional Empathy Scale (BEES; Italian adaptation: Meneghini et al., 2006) included 30 items measured on a seven-point Likert scale to assess empathy. The Interpersonal Reactivity Index (IRI; Italian adaptation: Ingoglia et al., 2016) measured the cognitive (Fantasy-F, Perspective Taking-PT) and affective (Empathic Concern-EC, Personal Distress-PD) components of empathy using 28 items on a seven-point Likert scale.

#### FFM Personality Traits

The Big Five Questionnaire-Short Form (BFQ-SF) developed by Caprara et al. (1993) assessed the five factors of personality: Extraversion, Agreeableness, Emotional stability, Conscientiousness, and Openness. The BFQ-SF consisted of 60 items scored on a five-point Likert scale.

#### Psychological Well-Being and Mental Health

The Subjective Happiness Scale (SHS; Italian adaptation: Iani et al., 2014) comprised four items measured on a seven-point Likert scale, assessing the level of life satisfaction. The Basic Psychological Needs Scale (BPNS; Italian adaptation: Szadejko, 2003) included 21 items measured on a five-point Likert scale, measuring autonomy, competence, and relatedness. The State-Trait Inventory of Cognitive and Somatic Anxiety (STICSA; Italian adaptation: Carlucci et al., 2018) consisted of 21 items measured on a four-point Likert scale, assessing anxiety characteristics. The Teate Depression Inventory (TDI; Balsamo & Saggino, 2013) comprised 21 items measured on a five-point Likert scale, evaluating depressive symptoms.

#### Disinhibition vs. Constraint

The Disinhibition vs. Constraint Inventory (DvC; Dindo et al., 2009) consisted of 65 items measured on a five-point Likert scale. The items were grouped into five subscales: prosociality, manipulativeness, distractibility, risk-taking, and orderliness.

#### Social Desirability

Social desirability was assessed using the Marlowe-Crowne (MC) scale-short form (Italian adaptation: Manganelli Rattazzi et al., 2000), which included nine items measured on a five-point Likert scale. Positive correlations of psychological variables with MC scores indicated an inclination to overestimate psychological traits, while negative correlations indicated a tendency to underestimate them. The assessment of social desirability aimed to evaluate the validity of individual responses on the SD3, DD, and other psychological scales, as previous research has shown that subjective ratings, particularly those related to the Dark Triad traits, could be influenced by this response set (Kowalski et al., 2016).

#### Statistical Analyses

##### Descriptive Analysis

Descriptive statistics, including the number of valid cases, means, standard deviations, skewness, and kurtosis, were calculated for each psychological scale. Skewness and kurtosis values within the range of ±2 were considered acceptable (Gravetter & Wallnau, 2014). Correlations between each psychological measure and the MC scale were examined to assess the impact of social desirability on subjective responses. Frequencies were calculated for sociodemographic variables.

##### Multi-Trait Multi-Method Analyses

To evaluate convergent and discriminant validity of DT traits measured by the SD3 and DD methods, we fit a six-factor Confirmatory Factor Analysis (CFA) model in which latent factors represented Machiavellianism, narcissism, and psychopathy measured by both the SD3 and DD. Each latent factor was defined by its respective items. Because these items were ordinal variables, diagonally weighted least squares (DWLS) was used for estimation. DWLS has no distributional assumptions and mitigates biases associated with Likert response scales (Rhemtulla et al., 2012). Model fit was examined using the Comparative Fit Index (CFI), Tucker–Lewis Index (TLI), Root Mean Square Error of Approximation (RMSEA), and Standardized Root Mean Square Residual (SRMR). Fit is considered excellent if CFI and TLI exceed .95 (acceptable if > .90), and good if RMSEA < .06 and SRMR < .08 (Schermelleh-Engel et al., 2003). The six-factor model assumed free covariances among latent variables. This allowed us to assess convergent and discriminant validity properties using the Heterotrait–Monotrait (HTMT) ratio and the Fornell–Larcker criterion, respectively. The HTMT ratio is computed as the average of heterotrait–heteromethod correlations (e.g., Machiavellianism items from the SD3 with psychopathy and narcissism items from the DD), divided by the average of monotrait–heteromethod correlations (e.g., Machiavellianism items measured by both SD3 and DD). Ideally, if two sets of measures are entirely independent, heterotrait–heteromethod correlations should be zero, while monotrait–heteromethod correlations should be one. Conversely, if the item sets measure the same construct, both types of correlations should be 1.00, resulting in an HTMT ratio of 1.00. In practical applications, an HTMT ratio threshold of .85 is recommended to determine whether two constructs are empirically distinct (HTMT < .85) or overlapping (HTMT ≥ .85) (Henseler et al., 2015). According to the Fronell-Larcker criterion (Voorhees et al., 2016) for assessing discriminant validity, each factor’s Average Variance Extracted (AVE)—a measure of how much variance in a set of observed indicators is explained by the latent construct—must exceed its shared variance with other factors, calculated as the square of their correlation. Specifically, if the square root of a factor’s AVE is greater than its correlation with any other factor, that factor is considered to have discriminant validity. Based on these analyses, we formally tested the convergent and discriminant validity of corresponding factors across the two methods by comparing modified models—where we constrained the correlations between identically labeled latent factors to 1.00—with the unconstrained model in which those correlations were freely estimated. These analyses were conducted using R software (https://cran.r-project.org) with the *lavaan* package (Rossel, 2012).

##### Nomological Analysis

To test the nomological consistency of SD3 and DD, a multifaceted analytic approach was followed, consisting of three steps (Thielmann & Hilbig, 2019). The Thielmann and Hilbig’s multifaceted method of nomological analysis follows a holistic approach by considering all relevant indicators that showed to be connected to the principal components of Dark Triad. This method refrains from pre-selecting criteria, through correlation matrices, and assures generalizability of outcomes in different contexts, reducing potential biases tied to a narrow selection of outcomes. The use of Bayesian regression models allows a more reliable selection of outcomes than null hypothesis significance test approach. This method is not exempt from limitations. In particular, because it is based on prior research, it is subjected to potential selection bias and to differences in inventory properties. In the first step, the difference between the mean absolute Fisher’s *z* values ($\Delta \bar{r}$) of zero-order correlations for each correlation matrix was calculated to identify significant convergences or divergences between Dark Triad traits measured by SD3 and DD and the target variables. A mean absolute difference in zero-order correlations greater than .10 may suggest the potential influence of additional factors, beyond those considered, in shaping the relationships between variables. Additionally, the absolute difference between the mean *R*^2^ value ($\Delta {\bar{R}}^{2}$) of regression models for prediction consistency of SD3 and DD and the Intraclass Correlation Coefficient (*r*_ICC_) was computed to analyze the correlation profiles for each equivalent trait indicator. In the second step, Fisher’s *z*-tests were conducted to compare correlation matrices pairwise and determine the number of comparisons resulting in significant differences between the correlation coefficients. In the third step, regression coefficients resulting from multiple regression analyses were estimated to predict each criterion using all Dark Triad trait indicators from the scales. Bayes factors (BF_01_) were calculated for each regression model across sets of criteria. BF_01_ values were classified into three groups: BF_01_ ≥ 3 indicated strong evidence in favor of the alternative hypothesis (H_1_), BF_01_ ≤ 1/3 indicated strong evidence in favor of the null hypothesis (H_0_), and 1/3 < BF_01_ < 3 indicated inconclusive evidence for H_0_ or H_1_. The interpretation of Bayesian regression analysis involved assessing the prediction validity of the regression model when a specific Dark Triad trait was omitted. Low BF_01_ values indicated the high importance of Dark Triad traits.

Nomological consistency of inventories did not have defined cutoffs. However, some criteria proposed by Thielmann and Hilbig (2019) were considered to indicate satisfactory nomological consistency: small differences in absolute correlation coefficients ($\Delta \bar{r}$ ≤ .10), small differences in the amount of explained variance ($\Delta {\bar{R}}^{2}$ ≤ 5%), strong correlations between correlation matrices (*r*_ICC_ ≥ .80), non-significant differences between correlation matrices (pairwise *z*-tests with *p* ≥ .05), and a substantial percentage of similar conclusions about null and alternative hypotheses in multiple regressions based on Bayes factor analysis (≥ 80%).

These analyses were conducted using R software (https://cran.r-project.org) with the *BayesFactor* (Morey et al., 2015) and *cocor* (Diedenhofen & Diedenhofen, 2016) packages.

## Results

### Descriptive Analysis

Descriptive statistics for each psychological scale, including means, standard deviations, skewness, kurtosis, Cronbach’s alpha values, and 95% confidence intervals for alphas, are provided in (Supplementary Table S1). The skewness and kurtosis values were acceptable for all variables. Cronbach’s alpha values were generally acceptable or good, except for the autonomy subscale of the BPNS scale and the secondary psychopathy subscale of the LSRP, which had alpha values slightly below .60 but still close to acceptable standards. Significant correlations with the MC scale indicated that subjective responses on the SD3 and DD subscales were influenced by social desirability, with correlations ranging from -.55 to -.19.

### Multi-Trait Multi-Method Analyses

Despite significant, χ^2^(687, *N* = 504) = 1801.18, *p* < .001, the unconstrained model, in which latent-factor correlations were freely estimated, had a good fit (CFI = .967; TLI = .965; RMSEA = .060, SRMR = .069). Across both methods (SD3 and DD), each trait—Machiavellianism, narcissism, and psychopathy—was measured by items with statistically significant loadings (all *p* < .001), demonstrating that the items generally captured their intended constructs (details in Supplementary Table S2).

In the SD3, Machiavellianism showed predominantly moderate to high factor loadings. Apart from Item i1 (λ = .067), all items loaded on between .433 and .810, with Item i4 displaying the strongest coefficient. For narcissism, most SD3 items loaded in the low-to-moderate range (.217–.642), with Item i14 as the highest. Similarly, psychopathy items ranged from .238 to .731, with Item i15 presenting the strongest loading. In the DD instrument, Machiavellianism was characterized by consistently strong loadings (λ = .778–.850), while narcissism (λ = .502–.819) and psychopathy (λ = .373–.786) also showed moderate to strong loadings. These results suggested some variation in how each method effectively measured the respective Dark Triad trait. Internal consistency indexes highlighted this variation (details in Supplementary Table S2). The ordinal alpha (α) values for the SD3 subscales ranged from .684 (narcissism) to .760 (Machiavellianism), whereas average variance extracted (AVE) values ranged from .207 (narcissism) to .302 (Machiavellianism). By contrast, the DD subscales showed higher ordinal alpha values, from .702 (psychopathy) to .892 (Machiavellianism), and higher AVE, ranging from .420 (psychopathy) to .676 (Machiavellianism). These findings indicated that while the SD3 subscales demonstrated acceptable internal consistency, the DD subscales generally yield stronger reliability and extracted a greater proportion of shared variance in measuring each Dark Triad trait.

Next, we examined the latent variable correlations. Each Dark Triad trait—Machiavellianism, narcissism, and psychopathy—correlated strongly across the two methods (SD3 and DD), in some cases showing an ostensible overlap. For Machiavellianism (*r* = .927) and psychopathy (*r* = .876), both methods showed strong convergence. In contrast, narcissism demonstrated weaker convergence, with a moderate correlation between SD3 and DD methods (*r* = .718). The Fronell–Larcker analysis revealed some differences in convergence and discriminant validity (details in Supplementary Table S3). While both methods showed strong convergence for Machiavellianism and psychopathy, the SD3 method struggled with discriminant validity, as its AVE square roots were consistently lower than correlations with other latent variables. For narcissism, the weaker convergence between SD3 and DD, coupled with the SD3 scale’s poor discriminant validity, highlighted the need for refinement in its measurement. Overall, the DD scale appeared to better differentiate the Dark Triad traits. This conclusion was further supported by the analysis of the HTMT ratios (details in Supplementary Table S3). The HTMT ratios for the same trait were understandably high for Machiavellianism and psychopathy (i.e., .888 and .877, respectively) indicating good convergent validity between methods. However, the HTMT ratio for narcissism equal to .722 was comparatively lower, raising some concerns about the convergent validity of narcissism across instruments.

We formally tested the overlap of DT traits by comparing the fit of the unconstrained model to constrained models where correlations between identically labeled latent factors were fixed at 1.00, indicating perfect similarity. For Machiavellianism, Δχ^2^(1, *N* = 504) = 13.42, *p* < .001, and psychopathy, Δχ^2^(1, *N* = 504) = 14.44, *p* < .001, there was a significant loss of fit compared to the unconstrained model; however, differences in RMSEA, SRMR, and CFI were null, and ΔTLI was negligible (.001 for both). For narcissism, the constrained model showed not only a significant loss of fit, Δχ^2^(1, *N* = 504) = 73.23, *p* < .001, but also appreciable declines in other fit indices, with increases in RMSEA (+.002) and SRMR (+.001) and decreases in CFI (-.002) and TLI (-.003). These findings, and those previously reported, aligned with the conclusion that while Machiavellianism and psychopathy showed strong similarity across SD3 and DD methods, narcissism exhibited notable differences between the two methods. While these conclusions capitalize on findings within the SEM framework, further evidence is needed, such as examining the nomological network of each trait to assess their relationships with external variables and clarify the distinctiveness of the constructs across measurement methods.

### Nomological Analysis

In the first step of the analysis, mean absolute differences in zero-order correlations ($\Delta \bar{r}$) were calculated for each set of criteria. The mean absolute difference in zero-order correlations for the Psychopathy and Empathy set was .080, for the FFM personality traits set was .074, for the Mental Health set was .315, and for the Disinhibition vs. Constraint set was .078. Table 1 presents the standardized coefficients and *R*^2^ values from linear regression models used to assess the prediction consistency of SD3 and DD scales.

**Table 1:** Regression Analysis for Prediction Consistency Between the SD3 and DD Subscales and the Four Sets of Criteria

Dark Triad Scales								
Set of criteria	SD3 Mach	SD3 Nar	SD3 Psych	SD3 R^2^	DD Mach	DD Nar	DD Psych	DD R^2^
*Psychopathy and Empathy*								
BEES	-0.189***	-0.070	-0.202***	0.143	-0.064	0.052	-0.452***	0.222
IRI F	0.026	-0.071	-0.023	0.006	0.219***	0.077	-0.361***	0.091
IRI EC	-0.155**	-0.045	-0.230***	0.131	-0.165**	0.118*	-0.383***	0.203
IRI PT	-0.087	-0.006	-0.154**	0.047	-0.137*	0.068	-0.189***	0.068
IRI PD	0.114*	-0.283***	-0.045	0.076	0.155**	-0.076	-0.247***	0.048
LSRP PP	0.331***	0.135***	0.371***	0.467	0.382***	0.066	0.331***	0.451
LSRP SP	0.121*	-0.260***	0.401***	0.194	0.277***	-0.104*	0.092	0.086
*FFM Personality Traits*								
BFQ-SF E	-0.068	0.513***	0.086	0.277	0.001	0.322***	0.064	0.124
BFQ-SF A	-0.330***	0.096*	-0.238***	0.219	-0.148**	0.031	-0.324***	0.170
BFQ-SF C	0.003	0.226***	-0.228***	0.064	-0.184**	0.261***	-0.114*	0.062
BFQ-SF Em-St	-0.071	0.204***	-0.212***	0.065	-0.065	-0.111*	0.039	0.020
BFQ-SF O	-0.027	0.144**	-0.046	0.017	-0.046	0.166**	-0.099	0.023
*Mental Health*								
SHS	-0.123*	0.375***	-0.020	0.120	-0.031	0.057	0.082	0.010
BPNS Aut	-0.167**	0.286***	-0.008	0.075	-0.226***	0.090	0.205***	0.040
BPNS Com	-0.128*	0.431***	-0.169***	0.159	-0.188**	0.247***	0.012	0.044
BPNS Rel	-0.186***	0.389***	-0.185***	0.151	-0.237***	0.169**	-0.018	0.042
STICSA	0.096	-0.241***	0.180***	0.069	0.115	0.021	-0.060	0.011
TDI	0.085	-0.333***	0.186***	0.102	0.105	-0.069	-0.032	0.006
*Disinhibition vs. Constraint*								
DvC Pros-total	0.064	0.198***	-0.320***	0.085	-0.280***	0.309***	-0.128*	0.104
DvC Distr	0.146**	-0.305***	0.277***	0.138	0.237***	-0.142**	0.055	0.050
DvC Manip	0.337***	0.128***	0.361***	0.458	0.497***	0.089*	0.233***	0.506
DvC Order	-0.029	0.083	-0.245***	0.058	-0.097	0.001	-0.141**	0.044
DvC Risk	-0.100*	0.148**	0.347***	0.143	0.021	0.048	0.245***	0.079

*Note*. Standardized coefficients and R2 values for each model are reported.

SD3 = Short Dark Triad; DD = Dirty Dozen; Mach = Machiavellianism; Nar = Narcissism; Psych = Psychopathy; BEES = Balanced Emotional Empathy Scale; IRI = Interpersonal Reactivity Index (subscale: F = Fantasy; PT = Perspective Taking; EC = Empathic Concern; PD = Personal Distress); LSRP = Levenson Self-Report psychopathy Scale (subscales: PP = Primary psychopathy; SP = Secondary psychopathy); BFQ-SF = Big Five Questionnaire Short Form (E = Extraversion, A = Agreeableness, C = Conscientiousness, Em-St = Emotional Stability, O = Openness); SHS = Subjective Happiness Scale; BPNS = Basic Psychological Needs Scale (subscales: Aut = Autonomy; Com = Competence; Rel = Relatedness); STICSA = State-Trait Inventory of Cognitive and Somatic Anxiety; TDI = Teate Depression Inventory; DvC = Disinhibition vs. Constraint Inventory (subscales: Pros-total = Prosociality total score, Distr = Distractibility, Manip = Manipulativeness, Order = Orderliness, Risk = Risk Taking.

*p < .05. **p < .01. ***p < .001.

The absolute difference in *R*^2^ values ($\Delta {\bar{R}}^{2}$) between SD3 and DD regression models for the Psychopathy and Empathy set of criteria was .015 (1.5%). For the FFM personality traits set, $\Delta {\bar{R}}^{2}$as .049 (4.9%). The Mental Health set showed a $\Delta {\bar{R}}^{2}$ value of .087 (8.7%), and the Disinhibition vs. Constraint set had a $\Delta {\bar{R}}^{2}$ value of .020 (2.0%).

Regarding the Intraclass Correlation Coefficient (*r*_ICC_), for the Psychopathy and Empathy set, *r*_ICC_ values were .993 for Machiavellianism, .854 for narcissism, and .943 for psychopathy. For the FFM personality traits set, *r*_ICC_ values were .944 for Machiavellianism, .786 for narcissism, and .937 for psychopathy. The Mental Health set showed *r*_ICC_ values of .960 for Machiavellianism, .208 for narcissism, and .444 for psychopathy. Finally, for the Disinhibition vs. Constraint set, *r*_ICC_ values were .959 for Machiavellianism, .872 for narcissism, and 0.976 for psychopathy.

The first step analysis revealed moderate prediction consistency between SD3 and DD in the sets of criteria related to psychopathy and empathy, FFM personality traits, and disinhibition vs. constraint. However, prediction consistency was not achieved for the Mental Health set, with the narcissism and psychopathy scales showing the most divergent predictions. Specifically, SD3 narcissism was a valid predictor for all psychological well-being variables, and SD3 Psychopathy yielded valid predictions for almost all well-being variables, except for SHS and BPNS autonomy. On the other hand, DD Narcissism was only a valid predictor for BPNS competence and relatedness, while DD Psychopathy was only a valid predictor for BPNS autonomy.

In the second step of the analysis, Fisher's *z*-tests were conducted to compare pairwise correlations. The *p* values of these tests for each set of criteria are presented in Table 2. Results indicated that narcissism showed the largest number of significant pairwise differences, particularly in the Mental Health criteria. This suggests a notable disparity between the correlation matrices of SD3 and DD Narcissism.

**Table 2:** P Values of Fisher’s Z-Tests for Pairwise Comparison of Independent Correlation Coefficients for the Four Sets of Criteria

	SD3 and DD Traits		
Set of criteria	Mach	Nar	Psych
*Psychopathy and Empathy*			
BEES	0.596	0.349	0.009^b^
IRI F	0.277	0.035^a^	0.006^b^
IRI EC	0.701	0.292	0.062
IRI PT	0.577	0.825	0.534
IRI PD	0.782	0.004^b^	0.116
LSRP PP	0.455	0.768	0.557
LSRP SP	0.642	0.016^a^	0.007^b^
*FFM Personality Traits*			
BFQ-SF E	0.361	0.001^b^	0.358
BFQ-SF A	0.049^b^	0.293	0.742
BFQ-SF C	0.311	0.656	0.724
BFQ-SF Em-St	0.921	< 0.001^b^	0.035^b^
BFQ-SF O	0.889	0.813	0.378
*Mental Health*			
SHS	0.453	< 0.001^b^	0.624
BPNS Aut	0.879	0.003^b^	0.114
BPNS Com	0.715	0.003^b^	0.234
BPNS Rel	0.917	< 0.001^b^	0.428
STICSA	0.822	0.001^b^	0.044^b^
TDI	0.782	0.001^b^	0.098
*Disinhibition vs. Constraint*			
DvC Pros-total	0.035^b^	0.928	0.365
DvC Distr	0.981	0.012^b^	0.090
DvC Manip	0.008^b^	0.168	0.365
DvC Order	0.495	0.172	0.522
DvC Risk	0.408	0.148	0.237

*Note.* SD3 = Short Dark Triad; DD = Dirty Dozen; Mach = Machiavellianism; Nar = Narcissism; Psych = Psychopathy; BEES = Balanced Emotional Empathy Scale; IRI = Interpersonal Reactivity Index (subscale: F = Fantasy; PT = Perspective Taking; EC = Empathic Concern; PD = Personal Distress); LSRP = Levenson Self-Report psychopathy Scale (subscales: PP = Primary psychopathy; SP = Secondary psychopathy); BFQ-SF = Big Five Questionnaire Short Form (E = Extraversion, A = Agreeableness, C = Conscientiousness, Em-St = Emotional Stability, O = Openness); SHS = Subjective Happiness Scale; BPNS = Basic Psychological Needs Scale (subscales: Aut = Autonomy; Com = Competence; Rel = Relatedness); STICSA = State-Trait Inventory of Cognitive and Somatic Anxiety; TDI = Teate Depression Inventory; DvC = Disinhibition vs. Constraint Inventory (subscales: Pros-total = Prosociality total score, Distr = Distractibility, Manip = Manipulativeness, Order = Orderliness, Risk = Risk Taking.

^a ^coefficient significant at < .05. ^b ^coefficient significant at < .01. ^c^ coefficient significant at < .001.

The third step analysis involved estimating Bayesian factors (BF_01_) for regression models when a particular Dark Triad trait was omitted. The results are presented in Table 3.

**Table 3:** Bayesian Factor Values (BF_01_) of the Regression Models in Relation to Omitted DARK TRIAD Traits

	Omitted Trait					
Set of criteria	SD3 Mach	SD3 Nar	SD3 Psych	DD Mach	DD Nar	DD Psych
*Psychopathy and Empathy*						
BEES	0.008	2.387	0.003	4.511	4.999	< 0.001
IRI F	4.743	1.916	4.843	0.007	2.283	< 0.001
IRI EC	0.074	4.673	< 0.001	0.101	0.487	< 0.001
IRI PT	1.594	5.941	0.107	0.471	2.832	0.014
IRI PD	0.630	< 0.001	4.554	0.230	2.204	< 0.001
LSRP PP	< 0.001	0.015	< 0.001	< 0.001	3.538	< 0.001
LSRP SP	0.402	< 0.001	< 0.001	< 0.001	0.956	1.464
*FFM Personality Traits*						
BFQ-SF E	3.401	< 0.001	1.826	7.283	< 0.001	3.359
BFQ-SF A	< 0.001	0.810	< 0.001	0.254	6.646	< 0.001
BFQ-SF C	6.268	< 0.001	0.001	0.059	< 0.001	0.654
BFQ-SF Em-St	2.571	0.001	0.003	3.129	0.701	4.278
BFQ-SF O	4.845	0.087	3.873	4.241	0.058	1.097
*Mental Health*						
SHS	0.407	< 0.001	6.702	4.719	3.147	1.794
BPNS Aut	0.046	< 0.001	6.398	0.006	1.494	0.005
BPNS Com	0.304	< 0.001	0.030	0.052	< 0.001	5.794
BPNS Rel	0.009	< 0.001	0.011	0.003	0.047	5.594
STICSA	1.240	< 0.001	0.023	0.970	5.018	3.012
TDI	1.842	< 0.001	0.013	1.277	2.438	4.515
*Disinhibition vs. Constraint*						
DvC Pros-total	3.153	0.002	< 0.001	< 0.001	< 0.001	0.364
DvC Distr	0.132	< 0.001	< 0.001	0.003	0.197	3.607
DvC Manip	< 0.001	0.032	< 0.001	< 0.001	1.056	< 0.001
DvC Order	5.291	1.473	< 0.001	1.679	5.915	0.212
DvC Risk	1.116	0.048	< 0.001	6.111	4.289	< 0.001

*Note.* SD3 = Short Dark Triad; DD = Dirty Dozen; Mach = Machiavellianism; Nar = Narcissism; Psych = Psychopathy; BEES = Balanced Emotional Empathy Scale; IRI = Interpersonal Reactivity Index (subscale: F = Fantasy; PT = Perspective Taking; EC = Empathic Concern; PD = Personal Distress); LSRP = Levenson Self-Report psychopathy Scale (subscales: PP = Primary psychopathy; SP = Secondary psychopathy); BFQ-SF = Big Five Questionnaire Short Form (E = Extraversion, A = Agreeableness, C = Conscientiousness, Em-St = Emotional Stability, O = Openness); SHS = Subjective Happiness Scale; BPNS = Basic Psychological Needs Scale (subscales: Aut = Autonomy; Com = Competence; Rel = Relatedness); STICSA = State-Trait Inventory of Cognitive and Somatic Anxiety; TDI = Teate Depression Inventory; DvC = Disinhibition vs. Constraint Inventory (subscales: Pros-total = Prosociality total score, Distr = Distractibility, Manip = Manipulativeness, Order = Orderliness, Risk = Risk Taking.

The findings clearly demonstrate that SD3 Narcissism is a crucial trait for the prediction validity of regression models for the Mental health criteria, while the same trait is less critical in the DD scale. The percentage of similar conclusions between SD3 and DD scales regarding the alternative hypothesis, based on BF_01_ values, was 38.1% for Psychopathy and Empathy criteria, 46.67% for FFM Personality Traits criteria, 38.89% for Mental Health criteria, and 46.67% for Disinhibition vs. Constraint criteria. These percentages are below the 80% threshold, indicating a substantial discrepancy between SD3 and DD scales in terms of consistency across all sets of criteria.

## Discussion and Conclusion

The present study examined the convergence and discriminant validity of two widely used measures of the Dark Triad (DT) traits—the Short Dark Triad (SD3) and the Dirty Dozen (DD)—using a Structural Equation Modeling (SEM) framework. Both the SD3 and DD scales demonstrate acceptable internal consistency in assessing Dark Triad traits and strong factor intercorrelations, consistent with previous research (Gamache et al., 2018; Geng et al., 2015; Maples et al., 2014; Schimmenti et al., 2019). Contrary to previous studies, which identified the SD3 scale as demonstrating greater validity (Gamache et al., 2018; Geng et al., 2015; Maples et al., 2014), our findings highlight the DD scale as the more robust instrument due to its superior item loadings, higher reliability indices, and greater Average Variance Extracted (AVE).

The findings revealed strong convergence for Machiavellianism and psychopathy across the two scales, as evidenced by high correlations between identically labeled factors. In contrast, narcissism demonstrated weaker convergence, with moderate correlations between the SD3 and DD scales. These results highlight important differences in the measurement focus of the two instruments, particularly for narcissism. The Fronell–Larcker analysis provided further evidence for the discriminant validity of the DT traits. While the DD scale consistently demonstrated acceptable discriminant validity, the SD3 scale exhibited significant overlap between Machiavellianism, psychopathy, and narcissism, suggesting challenges in distinguishing these constructs. Similarly, the heterotrait-monotrait (HTMT) ratios supported strong discriminant validity for DD but raised concerns about the SD3, particularly for narcissism.

Nomological network analyses further confirmed these findings by examining the relationships of DT traits with external constructs, including the Big Five, empathy, and mental health outcomes. There is convergence between SD3 and DD scales in relation to psychopathy and Machiavellianism, as they are negatively correlated with agreeableness and conscientiousness (Maples et al., 2014; O’Boyle et al., 2015; Vize et al., 2018). Notably, narcissism measured by SD3 and DD showed distinct patterns of association: SD3-Narcissism was more strongly linked to extraversion and openness, while DD-Narcissism showed stronger associations with neuroticism and lower empathy. These differences—yet observed in previous literature (Maples et al., 2014)—further underscore the divergent operationalizations of narcissism across scales. Literature reported that narcissism is not a single, cohesive trait but it presents multiple and variable aspects (Crowe et al., 2019). The SD3 appeared to focus predominantly on grandiose narcissism, emphasizing traits such as assertiveness and dominance, whereas the DD scale likely encompassed both grandiose and vulnerable aspects of narcissism. The distinct nomological profiles of these measures supported this interpretation.

In terms of mental health, our study replicated and extended previous research (Aghababaei & Błachnio, 2015), indicating that both Machiavellianism and psychopathy were negatively associated with positive functioning and well-being (i.e., subjective happiness, basic psychological needs satisfaction) and positively associated with negative outcomes (i.e., anxiety, depression). SD3 Narcissism demonstrated significant positive associations with measures of well-being and extraversion, consistent with previous research (Egan et al., 2014). In contrast, DD Narcissism did not show significant correlations with most measures of well-being, except for the BPNS competence and relatedness scale. These divergences further supported previous findings that SD3 and DD measure different forms of narcissism (Maples et al., 2014).

Regarding disinhibition and constraint, both SD3 and DD Psychopathy significantly predicted various aspects of disinhibition and prosocial attitudes. Machiavellianism and psychopathy positively predicted distractibility and manipulativeness, and negatively predicted prosociality. Narcissism showed positive correlations with prosociality and manipulativeness, and negative correlations with distractibility. These associations are consistent with the notion that individuals with negative personality traits are more prone to misconduct and substance abuse (Azizli et al., 2016; Stenason & Vernon, 2016).

Collectively, the findings of the present study highlighted a potential ‘jingle-jangle’ fallacy in measuring DT traits, which posits that similar labels (e.g., narcissism) may not represent identical constructs across different measures, and different labels (e.g., Machiavellianism and psychopathy) may sometimes overlap significantly. Furthermore, our findings underscore the need for researchers to carefully select DT scales based on their specific objectives and the construct clarity provided by each measure.

This study contributed methodologically by integrating a robust SEM framework with Fronell–Larcker and HTMT analyses to assess the validity of DT measures. These approaches provided a comprehensive evaluation of both convergence and discriminant validity, highlighting strengths and weaknesses in existing measures. Additionally, the inclusion of a nomological network analysis added depth to our understanding of how these traits were related to broader personality constructs and mental health outcomes. By comparing two prominent DT measures, this study offered valuable insights for both researchers and practitioners. Future research should focus on revising the SD3 items, particularly in Italian, as the observed lack of discriminant validity may arise from item heterogeneity.

Several limitations should be acknowledged. First, the reliance on self-report measures might have introduced some bias due to social desirability or response tendencies. Future research could complement self-reports with behavioral or observational methods to enhance validity. Second, the study was conducted on an Italian sample, which may limit generalizability to other cultural contexts. Given potential cultural variations in the expression of DT traits, replication in diverse populations is necessary. Third, the study’s design did not account for the measurement of vulnerable narcissism explicitly, which may partly explain the weaker convergence observed for this trait.

Notwithstanding limitations, overall, this study demonstrated that while the SD3 and DD scales are consistent in measuring Machiavellianism and psychopathy, their operationalizations of narcissism diverged significantly. The SD3’s challenges with discriminant validity highlight the need for careful scale selection depending on research objectives. By leveraging advanced psychometric methods and nomological network analyses, this study contributes to the ongoing refinement of DT measurement and emphasizes the importance of aligning measurement tools with theoretical clarity.

## Supplementary Materials

For this article, the following Supplementary Materials are available:

- Code. (Tommasi, 2025)
- Study materials. (Tommasi, 2025)

## Data Availability

For this article, codes and materials are available at Tommasi (2025).

## Appendix

**Table S1:** Descriptive Statistics of Psychological Scales Used in the Present Research

Psychological Scale	Subscale	# Valid Cases	*M*	*SD*	Skewness	Kurtosis	Cronbach's α	90% CI [LL,UL]	Correlation with MC	Prob. of Corr.
BEES		503	24.03	21.82	-.15	.10	.88	.87, .88	.32	< .001
IRI	F	503	14.68	4.04	-.01	-.27	.79	.78, .80	.00	.94
	PT	504	22.63	3.59	.01	-.66	.68	.66, .70	.36	< .001
	EP	502	23.56	4.52	-.20	-.04	.75	.74, .76	.29	< .001
	PD	503	19.03	4.97	.06	-.06	.80	.79, .81	-.01	.87
SHS		497	18.56	4.57	-.34	-.22	.71	.69, .73	.08	.09
STICSA		504	39.80	9.17	.41	-.32	.87	.86, .87	-.16	< .001
TDI		503	28.18	12.24	.35	-.01	.91	.90, .91	-.19	< .001
BPNS	Aut	504	24.93	3.56	-.46	.19	.54	.51, .56	.09	.06
	Com	504	20.78	3.71	-.10	-.34	.63	.61, .65	.14	< .01
	Rel	504	31.28	4.54	-.51	.32	.74	.72, .75	.28	< .001
LSRP	PP	501	30.99	6.64	.11	-.35	.82	.81, .83	-.42	< .001
	SP	502	21.58	3.66	-.01	.71	.56	.54, .58	-.32	< .001
SD3	Mach	504	24.16	5.79	.23	-.19	.72	.70, .73	-.44	< .001
	Nar	504	25.93	4.94	.24	.31	.64	.63, .66	-.19	< .001
	Psy	504	19.10	5.14	.36	-.27	.68	.66, .69	-.46	< .001
DD	Mach	504	8.86	4.92	1.09	.42	.83	.82, .84	-.55	< .001
	Nar	504	14.37	5.21	-.14	-.79	.74	.72, .75	-.37	< .001
	Psy	504	10.33	4.52	.71	.31	.62	.59, .65	-.41	< .001
BFQ-SF	E	503	37.83	5.81	.32	.38	.69	.68, .70	-.06	.15
	A	503	39.75	5.38	-.02	.11	.66	.65, .67	.44	< .001
	C	503	43.18	6.22	.05	-.08	.77	.76, .78	.17	< .001
	Em-St	503	34.54	7.67	.13	-.36	.84	.83, .84	.23	< .001
	O	503	40.79	6.76	-.09	-.18	.77	.76, .78	.09	< .05
DIS-I	Pros-Cons	502	3.74	.40	-.27	1.53	.71	.70, .72	.23	< .001
	Pros-Goal	504	3.80	.50	-.41	.64	.69	.68, .71	.34	< .001
	Pros	502	3.69	.50	-.22	.42	.66	.64, .68	.03	.57
	Distr	503	2.79	.64	.27	.09	.87	.87, .88	-.31	< .001
	Manip	503	2.27	.58	.33	.08	.83	.82, .83	-.52	< .001
	Order	502	3.42	.73	-.31	-.11	.84	.83, .85	.17	< .001
	Risk	504	2.85	.59	.37	.34	.69	.68, .71	-.10	< .05
	total	503	2.59	.29	.10	-.12	.81	.81, .82	-.46	< .001
MC		503	31.75	5.53	-.26	-.08	.68	.66, .69		

*Note.* Correlations with the Marlowe-Crowne scale with corresponding probabilities are also shown.

CI = confidence interval; BEES = Balanced Emotional Empathy Scale; IRI = Interpersonal Reactivity Index (subscale: F = Fantasy; PT = Perspective Taking; EP = Empathic Concern; PD = Personal Distress); SHS = Subjective Happiness Scale; STICSA = State-Trait Inventory of Cognitive and Somatic Anxiety;TDI = Teate Depression Inventory; BPNS = Basic Psychological Needs Scale (subscales: Aut = Autonomy; Com = Competence; Rel = Relatedness); LSRP = Levenson Self-Report psychopathy Scale (subscales: PP = Primary psychopathy; SP = Secondary psychopathy); SD3 = Short Dark Triad (subscales: Mach = Machiavellianism; Nar = Narcissism; Psy = Psychopathy); DD = Dirty Dozen (Mach = Machiavellianism; Nar = Narcissism; Psych = psychopathy); BFQ-SF = Big Five Questionnaire-Short Form (E = Extraversion;A = Agreeableness;C = Conscientiousness; Em-St = Emotional Stability; O = Openness); DIS-I = Disinhibition Inventory (subscales: Pros-Cons = Prosociality-Considerateness; Pros-Goal = Prosociality-Orientation Goal;Pros = Prosociality; Distr = Distractibility; Manip = Manipulativeness; Order = Orderliness; Risk = Risk Taking; total = DIS-I total score); MC = Marlowe-Crowne scale.

**Table S2:** Coefficients (β) and Relative Standard Errors (SE) for Baseline Model

Scale	Factor	Item	β	*SE*	*z* Value	*p*(*z*)	Residual
SD3	Mach	i4	0.810	0.015	52.605	<.001	0.344
		i1	0.067	0.017	3.963	<.001	0.996
		i7	0.433	0.015	28.746	<.001	0.812
		i10	0.473	0.015	30.759	<.001	0.776
		i13	0.725	0.015	49.810	<.001	0.475
		i16	0.614	0.015	40.667	<.001	0.623
		i19	0.447	0.015	29.257	<.001	0.800
		i22	0.476	0.015	31.258	<.001	0.773
		i25	0.565	0.015	37.483	<.001	0.680
	Nar	i2	0.496	0.021	24.093	<.001	0.754
		i5	0.569	0.021	27.289	<.001	0.676
		i8	0.510	0.021	24.647	<.001	0.740
		i11	0.418	0.020	21.200	<.001	0.825
		i14	0.642	0.021	29.922	<.001	0.588
		i17	0.370	0.020	18.516	<.001	0.863
		i20	0.441	0.020	21.947	<.001	0.806
		i23	0.265	0.020	13.299	<.001	0.930
		i26	0.217	0.021	10.477	<.001	0.953
	Psy	i3	0.477	0.017	28.788	<.001	0.773
		i6	0.336	0.016	21.094	<.001	0.887
		i9	0.554	0.017	33.415	<.001	0.693
		i12	0.378	0.016	23.173	<.001	0.857
		i15	0.731	0.017	41.954	<.001	0.466
		i18	0.238	0.017	13.828	<.001	0.943
		i21	0.300	0.019	15.483	<.001	0.910
		i24	0.584	0.018	32.604	<.001	0.659
		i27	0.678	0.017	39.830	<.001	0.541
DD	Mach	i1	0.841	0.013	66.159	<.001	0.294
		i4	0.817	0.013	62.285	<.001	0.332
		i7	0.778	0.013	60.424	<.001	0.394
		i10	0.850	0.013	67.393	<.001	0.277
	Nar	i3	0.644	0.017	37.712	<.001	0.586
		i6	0.502	0.016	30.544	<.001	0.748
		i9	0.729	0.018	41.367	<.001	0.468
		i12	0.819	0.019	43.697	<.001	0.329
	Psy	i2	0.373	0.017	21.844	<.001	0.861
		i5	0.613	0.018	34.997	<.001	0.624
		i8	0.740	0.018	41.769	<.001	0.452
		i11	0.786	0.018	43.208	<.001	0.383

*Note*. *z* values, *p*(*z*) values and residuals are also reported. Mach = Machiavellianism; Nar = Narcissism; Psy = Psychopathy.

**Table S3:** Factors Reliabilities (Coefficient Alpha, Coefficients Omega, Average Variance Extracted or AVE), Inter- and Intracorrelations Between the SD3 and the DD Factors (n = 504) and Heterotrait-Monotrait (HTMT) Matrix

Factors Reliabilities						
Subscale	α	α. ordinal	ω_1_	ω_2_	ω_3_	AVE
SD3-Mach	0.724	0.760	0.743	0.743	0.737	0.302
SD3-Nar	0.651	0.684	0.655	0.655	0.642	0.207
SD3-Psy	0.686	0.751	0.699	0.699	0.664	0.252
DD-Mach	0.845	0.892	0.853	0.853	0.854	0.676
DD-Nar	0.735	0.760	0.740	0.740	0.740	0.467
DD-Psy	0.617	0.702	0.691	0.691	0.705	0.420
Factor Correlations and AVE Square Root Values						
Subscale	SD3-Mach	SD3-Nar	SD3-Psy	DD-Mach	DD-Nar	DD-Psy
SD3-Mach	0.507^a^					
SD3-Nar	0.572	0.455^a^				
SD3-Psy	0.877	0.605	0.502^a^			
DD-Mach	0.927	0.500^b^	0.863	0.822^a^		
DD-Nar	0.696	0.718	0.628^b^	0.807	0.684^a^	
DD-Psy	0.744	0.505^b^	0.876	0.833	0.658	0.648^a^
HTMT Matrix						
SD3-Mach						
SD3-Nar	0.566					
SD3-Psy	0.751	0.543				
DD-Mach	0.888*****	0.390	0.694			
DD-Nar	0.678	0.722	0.377	0.737		
DD-Psy	0.731	0.562	0.877*****	0.792	0.518	

*Note.* SD3 = Short Dark Triad; DD = Dirty Dozen; Mach = Machiavellianism; Nar = Narcissism; Psy = Psychopathy.

All correlations are significant for *p* < .001. In factor correlations matrix, the square roots of AVE values are indicated by ^a ^, and are reported on the principal diagonal. Correlations lower than AVE square root values are indicated by ^b^. In HTMT matrix values > 0.85 are indicated by *.

## References

[r1] Aghababaei, N., & Błachnio, A. (2015). Well-being and the Dark Triad. Personality and Individual Differences, 86, 365–368. 10.1016/j.paid.2015.06.043

[r2] Azizli, N., Atkinson, B. E., Baughman, H. M., Chin, K., Vernon, P. A., Harris, E., & Veselka, L. (2016). Lies and crimes: Dark Triad, misconduct, and high-stakes deception. Personality and Individual Differences, 89, 34–39. 10.1016/j.paid.2015.09.034

[r3] Balsamo, M., & Saggino, A. (2013). *TDI-Teate Depression Inventory-Manuale [TDI-Teate Depression Inventory-Manual]*. Hogrefe.

[r4] Caprara, G. V., Barbaranelli, C., Borgogni, L., & Perugini, M. (1993). The “Big Five Questionnaire”: A new questionnaire to assess the five factor model. Personality and Individual Differences, 15, 281–288. 10.1016/0191-8869(93)90218-R

[r5] Carlucci, L., Watkins, M. W., Sergi, M. R., Cataldi, F., Saggino, A., & Balsamo, M. (2018). Dimensions of anxiety, age, and gender: Assessing dimensionality and measurement invariance of the State-Trait for Cognitive and Somatic Anxiety (STICSA) in an Italian sample. Frontiers in Psychology, 9, 2345. 10.3389/fpsyg.2018.02345 30538658 PMC6277473

[r6] Christie, R., & Geis, F. L. (1970). *Studies in Machiavellianism*. Academic Press.

[r7] Crowe, M. L., Lynam, D. R., Campbell, W. K., & Miller, J. D. (2019). Exploring the structure of narcissism: Toward an integrated solution. Journal of Personality, 87(6), 1151–1169. 10.1111/jopy.12464 30742713

[r8] Diedenhofen, B., & Diedenhofen, M. B. (2016). *Package ‘cocor’*. Comprehensive R Archive Network. https://cran. r-project. org/web/packages

[r9] Dindo, L., McDade-Montez, E., Sharma, L., Watson, D., & Clark, L. A. (2009). Development and initial validation of the disinhibition inventory: A multifaceted measure of disinhibition. Assessment, 16(3), 274–291. 10.1177/1073191108328890 19228581

[r10] Egan, V., Chan, S., & Shorter, G. W. (2014). The Dark Triad, happiness and subjective well-being. Personality and Individual Differences, 67, 17–22. 10.1016/j.paid.2014.01.004

[r11] Fleeson, W., & Noftle, E. E. (2009). In favor of the synthetic resolution to the person–situation debate. Journal of Research in Personality, 43(2), 150–154. 10.1016/j.jrp.2009.02.008

[r12] Gamache, D., Savard, C., & Maheux-Caron, V. (2018). French adaptation of the Short Dark Triad: Psychometric properties and a head-to-head comparison with the Dirty Dozen. Personality and Individual Differences, 122, 164–170. 10.1016/j.paid.2017.10.027

[r13] Geng, Y.-G., Sun, Q.-B., Huang, J.-Y., Zhu, Y.-Z., & Han, X.-H. (2015). Dirty Dozen and Short Dark Triad: A Chinese validation of two brief measures of the Dark Triad. Chinese Journal of Clinical Psychology, 23(2), 246–250.

[r14] Gore, W. L., & Widiger, T. A. (2016). Fluctuation between grandiose and vulnerable narcissism. Personality Disorders, 7(4), 363–371. 10.1037/per0000181 26986960

[r15] Gravetter, F., & Wallnau, L. (2014). *Essentials of statistics for the behavioral sciences* (8^th^ ed.). Wadsworth.

[r16] Henseler, J., Ringle, C. M., & Sarstedt, M. (2015). A new criterion for assessing discriminant validity in variance-based structural equation modeling. Journal of the Academy of Marketing Science, 43, 115–135. 10.1007/s11747-014-0403-8

[r17] Iani, L., Lauriola, M., Layous, K., & Sirigatti, S. (2014). Happiness in Italy: Translation, factorial structure and norming of the subjective happiness scale in a large community sample. Social Indicators Research, 118, 953–967. 10.1007/s11205-013-0468-7

[r18] Ingoglia, S., Lo Coco, A., & Albiero, P. (2016). Development of a Brief Form of the Interpersonal Reactivity Index (B–IRI). Journal of Personality Assessment, 98(5), 461–471. 10.1080/00223891.2016.1149858 27050826

[r19] Jonason, P. K., & Webster, G. D. (2010). The Dirty Dozen: A concise measure of the Dark Triad. Psychological Assessment, 22(2), 420–432. 10.1037/a0019265 20528068

[r20] Jones, D. N., & Paulhus, D. L. (2014). Introducing the Short Dark Triad (SD3): A brief measure of dark personality traits. Assessment, 21(1), 28–41. 10.1177/1073191113514105 24322012

[r21] Kowalski, C. M., Vernon, P. A., & Schermer, J. A. (2016). The general factor of personality: The relationship between the Big One and the Dark Triad. Personality and Individual Differences, 88, 256–260. 10.1016/j.paid.2015.09.028

[r22] Manganelli Rattazzi, A. M., Canova, L., & Marcorin, R. (2000). La desiderabilità sociale. Un’analisi di forme brevi della scala di Marlowe e Crowne [Social desirability. An analysis of brief forms of the Marlowe-Crowne’s scale]. TPM. Testing, Psychometrics, Methodology in Applied Psychology, 7, 5–17. https://www.tpmap.org/2000-vol-7-no-1-spring/

[r23] Maples, J. L., Lamkin, J., & Miller, J. D. (2014). A test of two brief measures of the dark triad: The dirty dozen and short dark triad. Psychological Assessment, 26(1), 326–331. 10.1037/a0035084 24274044

[r24] Marsh, H. W., Pekrun, R., Parker, P. D., Murayama, K., Guo, J., Dicke, T., & Arens, A. K. (2019). The murky distinction between self-concept and self-efficacy: Beware of lurking jingle-jangle fallacies. Journal of Educational Psychology, 111, 331–353. 10.25656/01:18125

[r25] Meneghini, A. M., Sartori, R., & Cunico, L. (2006). Adattamento e validazione su campione italiano della Balanced Emotional Empathy Scale di A. Mehrabian [Adaptation and validation of the A. Meherabian’s balanced emotional empathy scale on an Italian sample]. Ricerche di Psicologia, 1, 1000–1030.

[r26] Miller, J. D., & Lynam, D. R. (2015). Psychopathy and personality: Advances and debates. Journal of Personality, 83(6), 585–592. 10.1111/jopy.12145 25329442

[r27] Morey, R. D., Rouder, J. N., Jamil, T., & Morey, M. R. D. (2015). *Package ‘bayesfactor’*. Comprehensive R Archive Network. https://cran.r-project.org/package=BayesFactor

[r28] Muris, P., Merckelbach, H., Otgaar, H., & Meijer, E. (2017). The malevolent side of human nature: A meta-analysis and critical review of the literature on the Dark Triad (narcissism, Machiavellianism, and psychopathy). Perspectives on Psychological Science, 12(2), 183–204. 10.1177/1745691616666070 28346115

[r29] O’Boyle, E. H., Forsyth, D. R., Banks, G. C., Story, P. A., & White, C. D. (2015). A meta‐analytic test of redundancy and relative importance of the dark triad and five‐factor model of personality. Journal of Personality, 83(6), 644–664. 10.1111/jopy.12126 25168647

[r30] Paulhus, D. L., & Williams, K. M. (2002). The Dark Triad of personality: Narcissism, Machiavellianism, and psychopathy. Journal of Research in Personality, 36(6), 556–563. 10.1016/S0092-6566(02)00505-6

[r31] Rhemtulla, M., Brosseau-Liard, P. É., & Savalei, V. (2012). When can categorical variables be treated as continuous? A comparison of robust continuous and categorical SEM estimation methods under suboptimal conditions. Psychological Methods, 17(3), 354–373. 10.1037/a0029315.supp 22799625

[r32] Rossel, Y. (2012). lavaan: An R package for structural equation modeling. Journal of Statistical Software, 48(2), 1–36. 10.18637/jss.v048.i02

[r33] Schermelleh-Engel, K., Moosbrugger, H., & Müller, H. (2003). Evaluating the fit of structural equation models: Tests of significance and descriptive goodness-of-fit measures. Methods of Psychological Research Online, 8(2), 23–74. 10.23668/psycharchives.12784

[r34] Schimmenti, A., Jonason, P. K., Passanisi, A., La Marca, L., Di Dio, N., & Gervasi, A. M. (2019). Exploring the dark side of personality: Emotional awareness, empathy, and the Dark Triad traits in an Italian Sample. Current Psychology, 38(1), 100–109. 10.1007/s12144-017-9588-6

[r35] Somma, A., Fossati, A., Patrick, C., Maffei, C., & Borroni, S. (2014). The three‐factor structure of the Levenson Self‐Report Psychopathy Scale: Fool’s gold or true gold? A study in a sample of Italian adult non‐clinical participants. Personality and Mental Health, 8(4), 337–347. 10.1002/pmh.1267 25132649

[r36] Somma, A., Paulhus, D. L., Borroni, S., & Fossati, A. (2019). Evaluating the psychometric properties of the Short Dark Triad (SD3) in Italian adults and adolescents. European Journal of Psychological Assessment, 36(1), 185–195. 10.1027/1015-5759/a000499

[r37] Stenason, L., & Vernon, P. A. (2016). The Dark Triad, reinforcement sensitivity and substance use. Personality and Individual Differences, 94, 59–63. 10.1016/j.paid.2016.01.010

[r38] Szadejko, K. (2003). Percezione di autonomia, competenza e relazionalità. Adattamento italiano del questionario Basic Psychological Needs Scale [Perception of autonomy, competence and relatedness. Italian adaptation of the Basic Psychological Needs Scale]. Orientamenti Pedagogici, 50(5), 53–72. https://rivistedigitali.erickson.it/orientamenti-pedagogici/archivio/vol-50-n-5

[r39] Thalmayer, A. G., Saucier, G., & Eigenhuis, A. (2011). Comparative validity of Brief to Medium-Length Big Five and Big Six Personality Questionnaires. Psychological Assessment, 23(4), 995–1009. 10.1037/a0024165 21859221

[r40] Thielmann, I., & Hilbig, B. E. (2019). Nomological consistency: A comprehensive test of the equivalence of different trait indicators for the same constructs. Journal of Personality, 87(3), 715–730. 10.1111/jopy.12428 30218453

[r41] Tommasi, M. (2025). *EJOP* [OSF project page containing R program codes and study questionnaire]. OSF. https://osf.io/rbhyt

[r42] Vize, C. E., Lynam, D. R., Collison, K. L., & Miller, J. D. (2018). Differences among dark triad components: A meta-analytic investigation. Personality Disorders, 9(2), 101–111. 10.1037/per0000222 27736106

[r43] Voorhees, C. M., Brady, M. K., Calantone, R., & Ramirez, E. (2016). Discriminant validity testing in marketing: An analysis, causes for concern, and proposed remedies. Journal of the Academy of Marketing Science, 44(1), 119–134. 10.1007/s11747-015-0455-4

[r44] Watts, A. L., Waldman, I. D., Smith, S. F., Poore, H. E., & Lilienfeld, S. O. (2017). The nature and correlates of the dark triad: The answers depend on the questions. Journal of Abnormal Psychology, 126(7), 951–968. 10.1037/abn0000296 29106280

